# An Amperometric Biosensor for the Determination of Bacterial Sepsis Biomarker, Secretory Phospholipase Group 2-IIA Using a Tri-Enzyme System

**DOI:** 10.3390/s18030686

**Published:** 2018-02-26

**Authors:** Nik Nurhanan Nik Mansor, Tan Toh Leong, Eka Safitri, Dedi Futra, Nurul Saadah Ahmad, Dian Nasriana Nasuruddin, Azlin Itnin, Ida Zarina Zaini, Khaizurin Tajul Arifin, Lee Yook Heng, Nurul Izzaty Hassan

**Affiliations:** 1 School of Chemical Sciences and Food Technology, Faculty of Science & Technology, Universiti Kebangsaan Malaysia, Bangi 43600, Selangor DE, Malaysia; niknurhanan@gmail.com (N.N.N.M.); leeyookheng@yahoo.co.uk (L.Y.H.); 2 Department of Emergency Medicine, Faculty of Medicine, Universiti Kebangsaan Malaysia, Cheras 56000, Kuala Lumpur, Malaysia; sebastianttl@yahoo.co.uk (T.T.L.); saadah182@gmail.com (N.S.A.); ida_zarina74@yahoo.com (I.Z.Z.); 3 Department of Chemistry, Faculty of Mathematics and Natural Sciences, Syiah Kuala University (USK), Darussalam-Banda Acheh 23111, Indonesia; e.safitri@yahoo.co.id; 4 The Department of Chemistry Education, Faculty of Education, Universitas Riau, Kampus Binawidya KM 12.5, Pekanbaru 28293, Riau, Indonesia; futra.dedi@yahoo.com; 5 Department of Pathology, Faculty of Medicine, Universiti Kebangsaan Malaysia, Cheras 56000, Kuala Lumpur, Malaysia; dal.nuun@gmail.com (D.N.N.); nazlin@ppukm.ukm.edu.my (A.I.); 6 Department of Biochemistry Education, Faculty of Medicine, Universiti Kebangsaan Malaysia, Cheras 56000, Kuala Lumpur, Malaysia; ktarifin@yahoo.com

**Keywords:** amperometric biosensor, choline kinase, choline oxidase, horseradish peroxidase, sepsis, bacterial infection, sPLA2-IIA

## Abstract

A tri-enzyme system consisting of choline kinase/choline oxidase/horseradish peroxidase was used in the rapid and specific determination of the biomarker for bacterial sepsis infection, secretory phospholipase Group 2-IIA (sPLA2-IIA). These enzymes were individually immobilized onto the acrylic microspheres via succinimide groups for the preparation of an electrochemical biosensor. The reaction of sPLA2-IIA with its substrate initiated a cascading enzymatic reaction in the tri-enzyme system that led to the final production of hydrogen peroxide, which presence was indicated by the redox characteristics of potassium ferricyanide, K_3_Fe(CN)_6_. An amperometric biosensor based on enzyme conjugated acrylic microspheres and gold nanoparticles composite coated onto a carbon-paste screen printed electrode (SPE) was fabricated and the current measurement was performed at a low potential of 0.20 V. This enzymatic biosensor gave a linear range 0.01–100 ng/mL (*R*^2^ = 0.98304) with a detection limit recorded at 5 × 10^−3^ ng/mL towards sPLA2-IIA. Moreover, the biosensor showed good reproducibility (relative standard deviation (RSD) of 3.04% (*n* = 5). The biosensor response was reliable up to 25 days of storage at 4 °C. Analysis of human serum samples for sPLA2-IIA indicated that the biosensor has potential for rapid bacterial sepsis diagnosis in hospital emergency department.

## 1. Introduction

Bacterial sepsis can be described as a condition in which patients experience systemic inflammatory response syndrome associated with infection and one of the leading causes of death in Malaysia [1]. Currently available routine screening for bacterial sepsis include the white blood cell (WBC) count, erythrocyte sedimentation rate (ESR), and C-reactive protein (CRP), although these have poor sensitivity and specificity [2]. There is a need to distinguish biomarkers that are specific and sensitive for bacterial sepsis detection. Pierrakos and Vincent [3] reported that the Secretory Group IIA Phospholipase A2 (sPLA2-IIA) have high sensitivity and specificity in determining sepsis (~95%). This biomarker was also tested positive in humans for the diagnosis of septicemia [4,5,6].

sPLA2-IIA is an acute phase protein and participates in the host response to inflammation and generates inflammatory arachidonic acid metabolites [7]. The levels of sPLA2-IIA is effective in measuring systemic of inflammation in various bacteremic and non-bacteremic infections [8,9,10] and in differentiating between bacterial and viral infections [11]. Interestingly, the level of sPLA2-IIA correlate well with the severity of septic shock and its outcome [12,13]. High or markedly increase levels of sPLA2-IIA have also been shown to associate with adverse outcome in sepsis [14,15]. Rintala et al. [11] have proved that sPLA2-IIA reached maximal levels even at the beginning of the follow-up and suggested the cut-off value of 150 mg/L for sPLA2-IIA to identifying bacteremia within the first 24 h.

Current diagnosis of bacterial sepsis involves cumbersome and time-consuming methods of bacterial cultures or ELISA (enzyme-linked immunosorbent assay) in which antibodies produced towards the pathogen are measured. Thus, a rapid and simple technology to aid bacteria sepsis diagnosis will be useful. Enzyme based electrochemical biosensors have a potential to fulfill the requirements, through the development of portable instruments which is specific, fast and cost effective with sufficient sensitivity and selectivity to diagnose sepsis directly.

**Figure 1:**
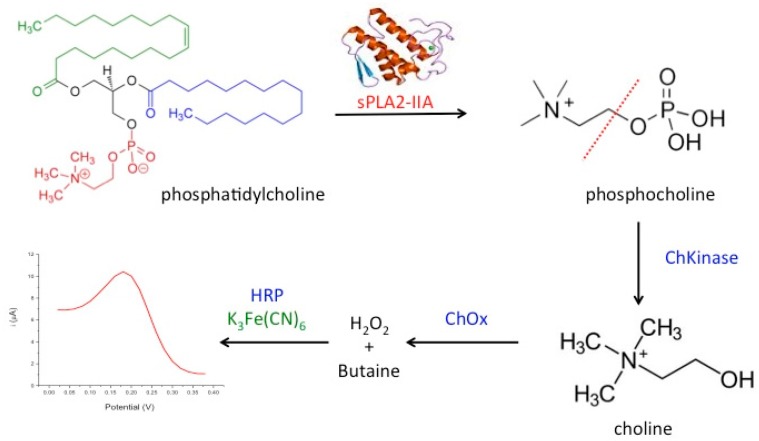
Design of a tri-enzyme system consisting of choline kinase (ChKinase)/choline oxidase (ChOx)/horseradish peroxidase (HRP) and potassium ferricyanide, K_3_Fe(CN)_6_ as mediator for the determination of the bacterial sepsis biomarker, sPLA2-IIA.

In the present work, we developed a novel amperometric biosensor for direct determination of the bacterial sepsis biomarker, sPLA2-IIA based on tri-enzyme system consisted of choline kinase (ChKinase), choline oxidase (ChOx) and horseradish peroxidase (HRP), which were covalently immobilized onto modified acrylic microspheres surface. The production of the enzyme sPLA2-IIA during sepsis conditions can be reacted with the substrate, phosphatidylcholine (PC) to form phosphocholine. Choline kinase (also known as choline phosphokinase) is an enzyme which catalyses the conversion of phosphocholine to choline. This is then acted upon by the second enzyme choline oxidase (ChOx) to produce hydrogen peroxide. Further, the third enzyme horseradish peroxidase (HRP) was used to react with hydrogen peroxide and this reaction is indicated by the redox mediator, potassium ferricyanide K_3_Fe(CN)_6_ operating at lower potential. The tri-enzyme system for sPLA2-IIA determination is summarized in Figure 1.

To fabricate an electrochemical biosensor, these enzymes were immobilized on acrylic microspheres functionalized with succinimide groups (NBA-NAS). The quantities of immobilized enzyme were optimized before immobilization onto the microspheres, independently. Thus, the amount of enzyme needed for an optimum detection system can be controlled by mixing the right weight of enzyme-conjugated microspheres before they were coated onto the electrode. Enzyme loaded microspheres were subsequently mixed with gold nanoparticles (AuNPs) to form a composite that was coated onto a carbon paste screen-printed electrode (SPE). The role of AuNPs is to improve the electrode conductivity as acrylic microspheres do not possess electrical conductivity. The use of acrylic microspheres modified with *N*-acryloxysuccinimide (NAS) for enzyme immobilization had been reported for amperometric capsaicin biosensor [16], potentiometric formaldehyde biosensor [17], and optical urea biosensor [18]. Enzymes deposited onto microspheres through covalent bonding have benefited from leaching and instability issue, hence enhance the overall performance.

## 2. Materials and Methods

### 2.1. Reagents and Solutions

All chemicals from commercial sources were of analytical grade and used as obtained. Choline oxidase from *Alcaligenes* sp., peroxidase from horseradish in lyophilized powder, approximately 150 U/mg and potassium ferricyanide, K_3_Fe(CN)_6_ were purchased from Sigma-Aldrich (Saint Louis, MO, USA). The source of sPLA2-IIA is honey bee venom (*Apis mellifera*), salt-free in lyophilized powder, 600–2400 U/mg protein and the substrate, L-a-phosphatidylcholine was obtained from egg yolk, type XVI-E (≥99%), 100 mg in lyophilized powder, ultrapure grade and kept in phosphate buffered saline (Sigma). Choline chloride 99%, Choline Kinase-a Inhibitor (CK37), 5 mg, was obtained from Merck. Potassium chloride (KCl), sodium hydroxide (NaOH), sodium acetate, sodium phosphate, *n*-butyl acrylate (NBA), 2-2-dimethoxy-2-phenylacetophenone (DMPP), 1,6-hexanediol diacrylate (HDDA), gold nanoparticles (AuNPs) and tris-hydroxymethyl aminomethane (Tris-HCl) were also supplied by Sigma-Aldrich). *N*-acryloxysuccinimide (NAS), sodium dodecyl sulfate (SDS) and ethanol 95% were from Systerm while hydrochloride acid 37% (HCl) from Riedel-de Haen. Phosphate buffer solution (PBS) was prepared using potassium dihydrogen phosphate and dipotassium hydrogen phosphate (Systerm) to achieve concentration of 0.1 M at pH 7. Preparation of standard solutions and cleaning laboratory glassware were performed using deionized water.

### 2.2. Apparatus and Electrode Deionized Water Was Used

Differential pulse voltammetry (DPV) experiments were operated using potentiostat, Autolab PGSTAT 12 (Metrohm, Herisau, Switzerland) at 0.02 mV step potential in the scanning range of −0.4–0.4 mV. SPE (Scrint Technology (M) Sdn. Bhd., Penang, Malaysia) modified with acrylic microspheres and gold nanoparticles (AuNPs) was used as working electrode. Meanwhile, a glassy carbon electrode and Ag/AgCl electrode were used as auxiliary and reference electrodes, independently. KCl solution of 3.0 M was utilized as the internal solution of the Ag/AgCl whereas all potential measured in this study were later referred to Ag/AgCl electrode. Homogeneous mixture of acrylic spheres was produced using sonicator bath, model Elma S30H and followed by centrifugation at 4000 rpm for 30 min (Centrifuge, HERMLE Z230A, 5500 rpm). These microspheres were washed three times using 5 × 10^−2^ M K-phosphate buffer (pH 7.0) followed by air-drying at ambient temperature. The morphology of poly(NBA) microspheres were analyzed using scanning electron microscope (SEM, LEO 1450VP, 20 kV acceleration voltage).

### 2.3. Immobilization Matrix—Acrylic Microspheres

NBA monomer (7 mL, SDS (0.01 g), HDDA (450 μL), DMPP (0.1 g), NAS (6 mg) and deionized water (15 mL) were mixed in a volumetric flask. At the end of sonication (10 min), the resulting mixtures turned milky white and was subsequently photocured for 600 s under continuous nitrogen gas purging in an ultraviolet exposure unit (R.S. Components Ltd., Northants, UK) of 15 Watt light intensity at 350 nm wavelength. Poly(NBA-NAS) microspheres were later collected by centrifugation (4000 rpm) for 30 min and washed repeatedly with potassium phosphate buffer solution of 5 × 10^−2^ M (pH 7) and dried at room temperature. The clean microspheres were stored at 4 °C when not in use.

### 2.4. Preparation of Modified SPEs

**Figure 2:**
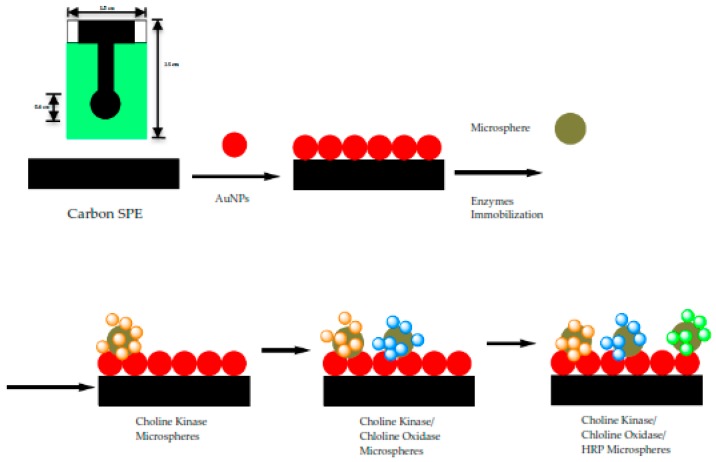
The fabrication procedure for amperometric biosensor for the determination of sPLA2-IIA as an indication of sepsis conditions. Three enzymes are used to determine the sPLA2-IIA using cascading enzymatic reactions. These enzymes are immobilized onto acrylic microspheres, (NBA-NAS), which formed a composite with gold nanoparticles (AuNPs) to improve the electrode conductivity. The composite is later deposited onto screen-printed electrode (SPE).

Screen-printed electrodes (SPEs) were cut into strips of 2.0 cm × 0.6 cm. The SPEs were modified with gold nanoparticles (1 mg AuNPs was dissolved in 300 µL EtOH) and this was accomplished by drop coating them on the SPEs. The three enzymes i.e., Horseradish peroxidase (4 µL), choline oxidase (8 µL) and choline kinase (4 µL) (All the enzymes = 0.5 mg enzymes in 100 µL potassium phosphate buffer solution of 0.01 M at pH 7) were immobilized separately on acrylic microspheres (1 mg microsphere was mixed in 100 µL potassium buffer solution of 0.01 M at pH 7) for 24 h. After overnight immobilization, all the mixture of acrylic microsphere (ACMS)-enzymes were centrifuged at speed: 10,000 rpm; duration: 10 min; temperature: 4 °C and washed with PBS solution 0.01 M pH 7 for three times. Each enzyme-conjugated microsphere was suspended in buffer solution again before carefully pipetted and dropped onto the confined area of the SPEs. The solution was left to dry on the working electrode up to 2 h before proceeding with the analysis. The modified SPEs were stored at chill temperature of 4 °C. The fabrication procedure of the biosensor is as shown in Figure 2.

### 2.5. Optimization of Bacterial Sepsis Biosensor

The response of bacterial sepsis biosensor was optimized to achieve the optimum working conditions for sepsis determination. The AuNps deposition was optimized from 0.03 to 0.1 mg, whilst acrylic microspheres (ACMS) were loaded between 0.02 and 0.2 mg onto the AuNPs-modified SPE surface. Effect of buffer type to the enzyme biosensor was investigated by using K-phosphate, tris-HCl, Na-phosphate and Na-acetate buffer. pH effect on the bacterial sepsis biosensor were carried out by varying the electrolyte pH from 5.0 to 9.0. Ratio of enzyme (HRP:Chox:ChKinase) was loaded between 0:0:0 and 2:1:1 mg on the ACMS-AuNPs-SPE electrode. For the optimization of the response time, the designed enzyme biosensors were dipped into a solution that contained the sPLA2-IIA enzyme at a fixed concentration from 0 to 30 min.

### 2.6. Selectivity Response

The selectivity response towards the biosensor was carried out with five metabolite compounds that can be found in human body fluids (e.g., serum, whole blood, etc.) at concentrations of 40 ng/mL and 80 ng/mL to match their concentrations in the diluted serum samples used for the tests later. The chosen metabolite compounds were urea, ascorbic acid, glucose, sucrose and citric acid. The selectivity study was carefully conducted via dipping the enzyme biosensor into the pure metabolite solutions alone and the DPV peak current response was recorded immediately. The biosensor responses obtained from these metabolite compounds were then compared to the response of the analyte sPLA2-IIA alone.

### 2.7. Amperometric Measurement

In order to detect sPLA2-IIA found in bacterial infection and sepsis, a potentiostat is used to record the electrochemical measurements through three electrode system developed consists of SPE as working electrode, a platinum electrode as counter electrode and a Ag/AgCl as reference electrode, respectively. Amperometric measurements are performed using the enzyme modified-SPEs as electrochemical transducer connected to a potentiostat (Metrohm Autolab B.V.) to generate electric current, which correlated to the enzymatic reaction progress and concentration of hydrogen peroxide, specifically. The response of the enzyme biosensor was obtained in a series of sPLA2-IIA enzyme solution from 0.01 ng/mL to 100 ng/mL where a linear calibration curve and the lowest detection limit were determined. The modified-SPEs were incubated for 10 min in 4 mL of solution containing PBS buffer, sPLA2-IIA enzyme and its substrate phosphatidylcholine. After incubation, 1 mL K_3_Fe(CN)_6_ (1 mM) was added and further incubated of another 5 min before the current response parallel to the enzymatic reaction was measured between the potential from −0.4 V to 0.4 V, where peak current was registered at 0.2 V.

### 2.8. Human Blood Serum Analysis

Human blood serum was analyzed to verify the accuracy and validity of sepsis biosensor based on developed amperometric method. These samples were collected using sterilized plastic bottles and kept frozen at all times. Serum collection was fully handled by Universiti Kebangsaan Malaysia Medical Centre (UKMMC). PBS buffer solution was added into serum samples to obtain 300 times dilution before measurement and kept the similar matrix conditions as KCl standard solutions. The samples were categorized into three categories, which were healthy samples, sepsis samples and septic shock samples. These diluted samples were analyzed according to the procedure mentioned in 2.7 using the biosensor.

The serum samples collected were evaluated using an ELISA test kit (Enzyme Immunometric Assay Kit; 585000, Cayman Chemical, Ann Arbor, MI, USA), concurrently. Each well of the plate has been coated with a monoclonal antibody specific for sPLA2, known as sPLA2 capture antibody which bind to any sPLA2-IIa introduced into the well. An Acetylcholinesterase:Fab’ Conjugate (AChE:Fab’) binds selectively to a different epitope on the sPLA2 molecule and form a ‘sandwich’ by linking on the opposite sides of the sPLA2 molecule.

These “sandwiches” were later deposited on the plate so the excess reagents would be washed away. Ellman’s Reagent was added to each well and the concentration of the analyte was determined by measuring the enzymatic activity of the AChE. The AChE:Fab’ was specific for sPLA2, and will not cross-react with other types of sPLAs, or other inflammatory mediators such as interleukin-1, platelet activating or tumor necrosis factor. The product of the AChE-catalyzed reaction has a distinct yellow color, which absorbs strongly at 412 nm. The intensity of this color was determined spectrophotometrically and proportional to the amount of bound conjugate, thus was also correspond to the concentration of sPLA2. The concentration of sPLA2 in the serum was tested as triplicates, indicated as pg/mL and determined against the standard curve of each ELISA assay. The ELISA kit has a minimum detectable concentration of 15.6 pg/mL.

## 3. Results and Discussion

### 3.1. FESEM Measurement of ACMS and Modified SPEs

**Figure 3:**
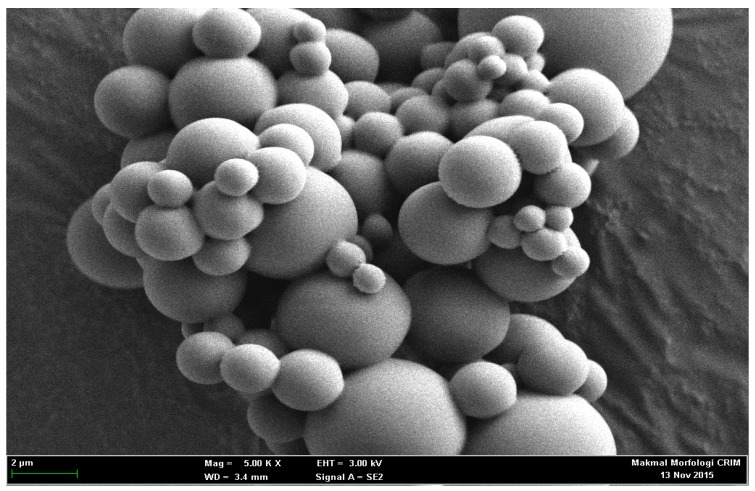
FESEM image of acrylic microsphere (scale of 1 cm = 2 µm, Mag = 5.0 K X, WD = 3.4 mm, EHT = 3.0 kV, Signal A = SE2).

Scanning electron micrograph (Figure 3) measured the average size of ACMS, approximately from 1.0 to 25 μm (54%), with 5.0 μm as the dominant diameter size. The size distribution of the microspheres will assure substantial enzyme binding capacity owed to its similar surface area of each microsphere, thus making the biosensor more sensitive [19].

**Figure 4:**
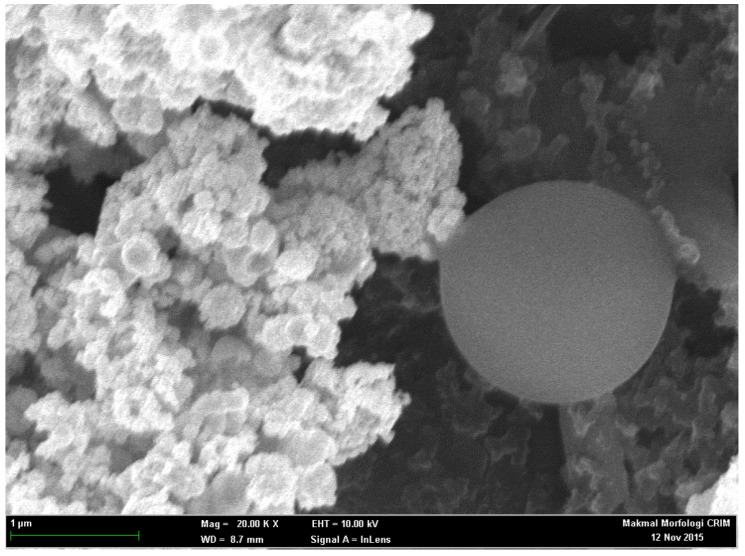
FESEM image of modified SPEs in the presence of AuNPs/NBA-NAS/respective enzymes (scale of 2 cm = 1 µm, Mag = 20 K X, WD = 8.7 mm, EHT = 10 kV, Signal A = InLens).

The microspheres were subsequently modified in the presence of AuNPs/NBA-NAS/tri-enzyme (ChKinase/ChOx/HRP) through covalent binding of highly reactive succinimide functional groups towards amino groups of the respective enzymes (Figure 4).

**Figure 5:**
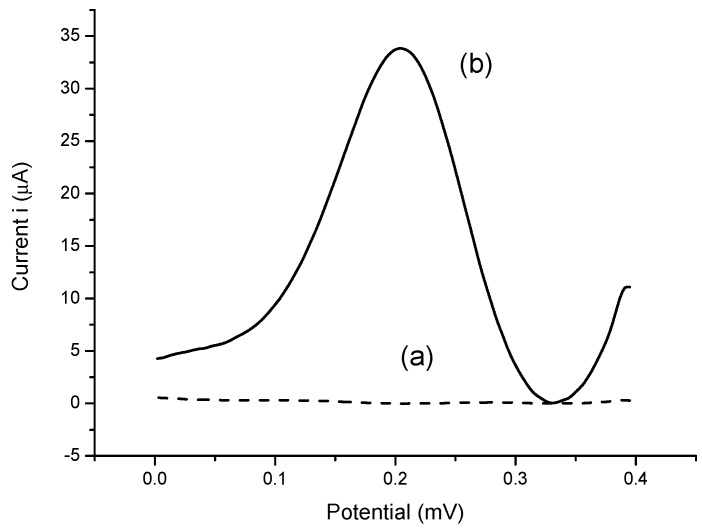
Differential pulse voltammograms of (**a**) bare electrode (**b**) modified HRP/NBA-NAS/AuNPs/SPE electrode. The experiment was conducted in H_2_O_2_ solution with 1.0 × 10^−6^ M concentration.

The differential pulse voltammograms of bare electrode and modified SPEs/nBA-NAS/AuNPs using HRP enzyme that give response in H_2_O_2_ solution with 1.0 × 10^−6^ M concentration. The differential pulse voltammograms (DPV) of electrodes were obtained between −0.4 and 0.4 mV. The signal showed the significant current change discovered between experiment (a) and (b). The HRP was successfully immobilized onto the acrylic microspheres, whereas the succinimide group of acrylic microsphere and amine group of HRP enzyme were bonded through covalent bond. Thus, there was no peak in experiment (a) as no electron conductivity has taken place. Without the inclusion of gold nanoparticles, acrylic microspheres and HRP enzyme on the SPEs, no current response was observed. Ulianas et al. [20] has observed an increase in the electron transfer rate when AuNPs were deposited on the SPE, corroborating that AuNPs as an effective electron transfer material as acrylic microsphere were non-conductive. The remaining two enzymes were modified in the same manner to the SPEs, which resulted in the same observation (Figure 5).

### 3.2. The Optimization of Bacterial Sepsis Biosensor

The amount of AuNPs was initially optimized to produce the best bacterial sepsis biosensor response performance via the hydrophobic and non-conductive microspheres. The optimized amount AuNPs and ACMS loading on the enzyme biosensor response is shown in Table 1. The biosensor response elevated with the increasing AuNPs loading from 0.03 to 0.05 mg, due to the increasing electrons transfer between AuNPs through the ACMS matrix. When the AuNPs amount exceeds 0.05 mg, the enzyme biosensor response declined due to the surplus coverage of AuNPs on the SPE surface, which limits electron transfer, resulting in low response of the biosensor. Rosmawani et al. [16] recently reported similar observation, in which the gold nanoparticles are capable to retain the bioactivity of the enzymes by providing stable immobilization on the homologous ACMS for their development on amperometric capsaicin biosensor. 

**Figure 6:**
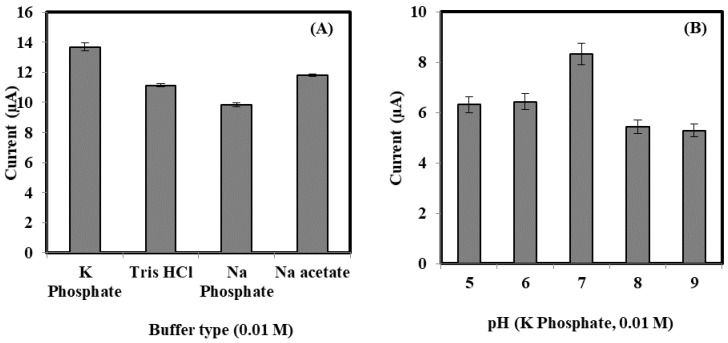

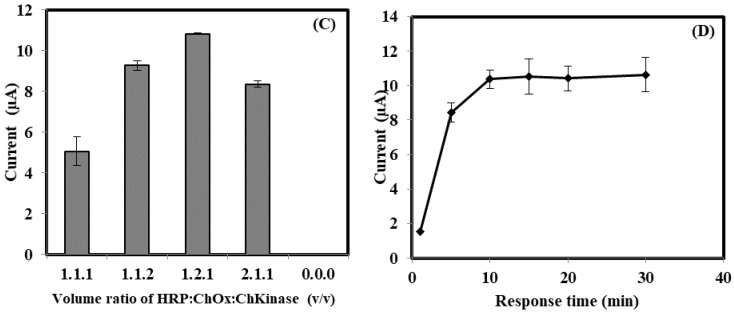
(**A**) Effect of the buffer type; (**B**) pH of the buffer; (**C**) volume ratio of HRP:ChOx:ChKinase and (**D**) response time toward the DPV current of the enzyme biosensor in the presence of 80 ng/mL sPLA2-IIA.

The effect of different buffer type toward the tri-enzyme biosensor response was shown in Figure 6A. The DPV peak current response in K-phosphate buffer was found to be higher compared to the DPV peak response obtained in the buffer of Tris-HCl, Na-acetate, and Na-phospate. The positive response in the K-phosphate buffer was contributed by neutral properties and favourable protection of K-phosphate buffer to the active biological cell and protein surface. Hence, excellent biosensor response at K-phosphate buffer was used to prepare the bacterial sepsis biosensor. The effect of pH on the biosensor responses was calculated in the range of pH 5.0–9.0 (Figure 6B). The current response increased with the increasing pH from 5.0 to 7.0. At pH approaching 7, the biosensor gave optimal response, which implies that pH 7.0 was sufficient for covalent bonding between the enzymes’ amino group and the succinimide ester of NAS. Thus, buffer at pH 7 is advisable to operate bacterial sepsis biosensor. At pH above 7, an overall decrease in the response was associated with the deactivation of enzymes. Furthermore, alkaline condition would impair the electrode. Similar trend was described by other groups when using HRP for determination of H_2_O_2_ [21,22,23] in the fabrication of sensor.

Effect of the volume ratio of HRP:ChOx:ChKinase can be seen as the biosensor signal increased progressively with increasing volume ratio of HRP:ChOx:ChKinase from 1:1:1 to 1:2:1 *v*/*v* (Figure 6C), due to increment quantities of enzyme (ChOx) attached to the acrylic-modified AuNPs-SPE surface. The amperometric biosensor response decreased with the changing volume ratio of HRP:ChOx:ChKinase from 1:2:1 to 0:0:0 (*v*/*v*) (Figure 6C), indicating decreased loading of enzyme (ChOx) bonded to the acrylic-AuNPs-SPE surface. Therefore, a volume ratio of 1:2:1 *v*/*v* was taken as for the enzyme biosensor.

The response time of the bacterial sepsis biosensor was estimated by exposing the biosensor to different concentrations of sPLA2-IIA at a fixed time from 5 to 30 min before measurements (Figure 6D). The DPV current response accordingly with the increasing incubation time from 0 to 10 min, indicating the collective reaction sPLA2-IIA with the tri-enzyme system. Meanwhile, the current signal stabilized consistently after 10 min owed to the full attachment to the active site of the enzyme.

### 3.3. Selectivity Study

To determine the specificity of the bacterial sepsis biosensor towards spLA2-IIA, the interference of important metabolite typically found in human body fluids such as urea, ascorbic acid, glucose, sucrose and citric acid were investigated (Table 2). The DPV current for the sPLA2-IIA enzyme was discovered to be higher than other metabolite compounds. The peak current percentages of ascorbic acid were found to be 19.6–18.4%, whilst urea compound demonstrated in significant response to the sepsis biosensor with peak current percentages between 11.4 and 10%. The peak current percentages for sucrose, glucose and citric acid were below 10%. Thus, the proposed sepsis biosensor showed selectivity towards sPLA2-IIA with maximum response.

### 3.4. Performances of Bacterial Sepsis Biosensor

**Figure 7:**
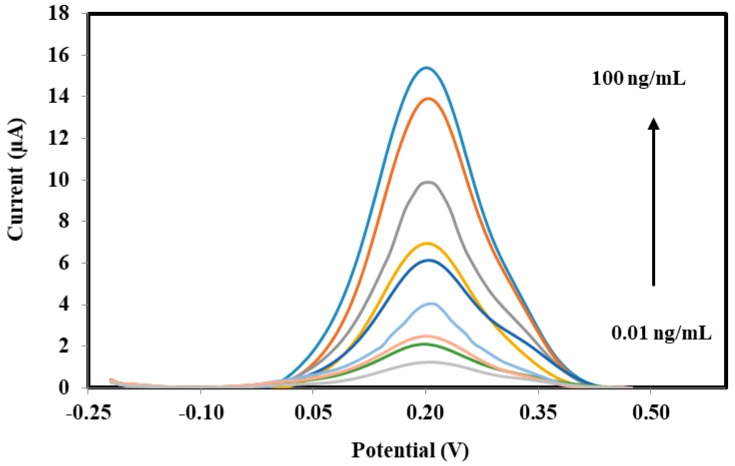
Differential pulse voltammograms of the electrochemical biosensor SPE/AuNPs/NBA-NAS/HRP:ChOx:ChKinase procured from various concentrations of human sPLA2-IIA 0.01–100 ng/mL in 1 mM K_3_Fe(CN)_6_, 0.01 M PBS pH 7.0 and KCl 0.1 M.

The differential pulse voltammograms (DPV) response proportionally increased with increasing concentration of human serum sPLA2-IIA (Table 3 & Figure 7), due to the electro-hydrolysis oxidation of K_3_Fe(CN)_6_ during the enzymatic conversion of phosphatidylcholine to H_2_O_2_ molecule whilst the inorganic compound redox reaction. Hence, it seemed possible to quantity H_2_O_2_ concentration indirectly by measuring the increased current of the K_3_Fe(CN)_6_ oxidation.

**Figure 8:**
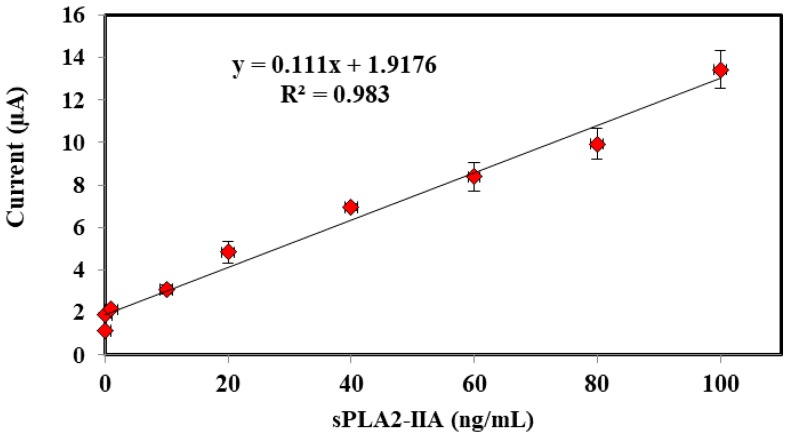
Calibration curve showed a linear relationship for current against various concentrations of human sPLA2-IIA 0.01–100 ng/mL in 5 mM K_3_Fe(CN)_6_, 0.01 M PBS pH 7.0 and KCl 0.1 M.

The calibration curve displayed a linear relationship for current (μA) signal against various concentrations of sPLA2-IIA in the range of 0.01–100 ng/mL (*R*^2^ = 0.98304). The detection limit is determined based on three times standard deviation of the biosensor response at the response curve approximating the limit of detection divided by linear calibration slope [24]. The limit detection of the proposed enzyme biosensor was estimated to be about 5 × 10^−3^ ng/mL (Figure 8). The excellent performances of the biosensor response probably originated from the highly monodispersed, high surface area and homogenous micro-sized microspheres of ACMS-modified gold nanoparticles that contributed to good diffusion layer on the surface electrode.

**Figure 9:**
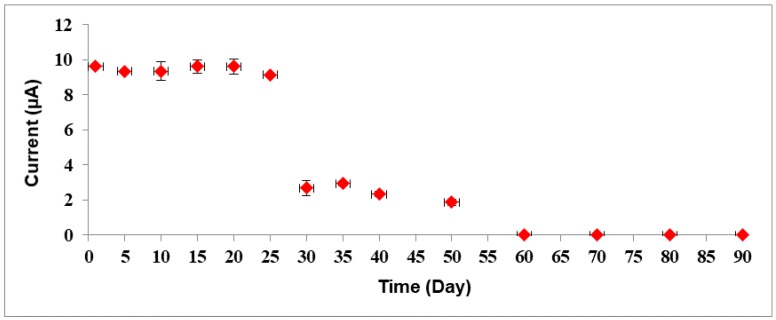
Effect of shelf life towards bacterial sepsis biosensor response. The biosensor was stored in 0.05 M K-phosphate buffer pH 7.0 at 4 °C when not in use.

The reproducibility of the biosensor was produced based on five variation batches of sensor responses, whereas each batch measurement was repeated three times for all concentrations of standard solutions. The reproducibility was determined with relative standard deviations (RSD) of 3.04% (*n* = 5). The shelf life of bacterial sepsis biosensor was later observed using a different biosensor electrode for each measurement (data not shown). Hence, several electrode biosensors were assembled and kept in K-phosphate buffer at 4 °C before being tested every five days before the biosensor responses start to deteriorate (Figure 9). The biosensor response was found stable up to 20 days of storage period with satisfactory response retained between 90% to 95% when compared to its response on the first day. After 20 days of storage, the biosensor response diminished up to 20% to 30% between 30 and 50 days of storage period. Narang et al. [25] found that their multienzyme electrode can be used over 40 days with minimal loss when stored at 4 °C.

### 3.5. Validation of the Sepsis Biosensor Using Human Serum Smples

The data of DPV peak current toward various human sPLA2-IIA was triplicate and the detection were verified by potassium ferricyanide, K_3_Fe(CN)_6_ as mediator (Table 4).

The standard deviation (SD) and relative standard deviation (RSD) of human sPLA2-IIA level for three samples of human serum (Healthy, Sepsis and Septic Shock) was showed in Table 5.

**Figure 10:**
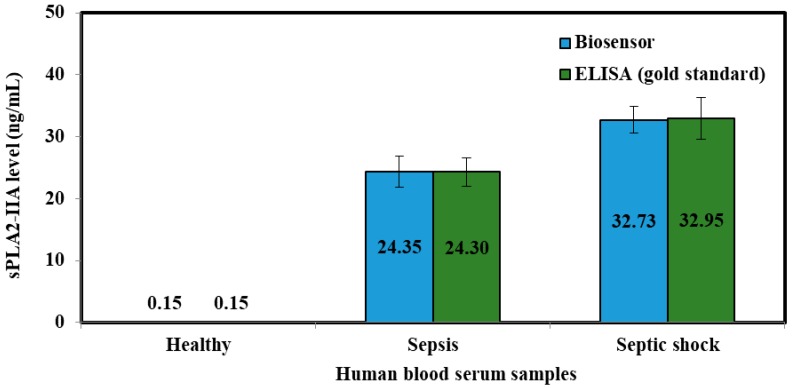
Comparison between tri-enyzme biosensor (blue bar chart) and ELISA method (green bar chart) shows that the biosensor prototype has similar ability to distinguish sepsis severity.

**Figure 11:**
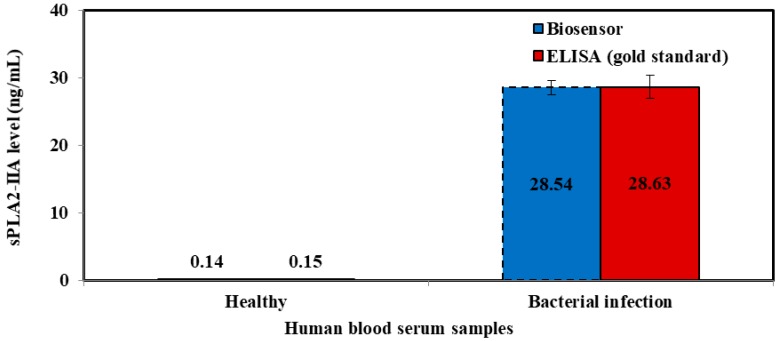
Comparison between tri-enyzme biosensor (blue bar chart) and ELISA method (red bar chart) shows that biosensor prototype has similar ability to distinguish bacterial infection.

Evaluation of the sPLA2-IIA in the human blood serum samples were achieved by both biosensor and ELISA immunoassay method (Figure 10 and Figure 11). The resulting biosensor demonstrated that the relative standard deviation (RSD) and standard deviation (SD) were ≤10% and (0.01–2.46), respectively. While, ELISA immunoassay methods showed that the ≤10% for RSD and (0.01–3.37) for SD was achieved. Based on the RSD and SD values obtained from both biosensor and ELISA methods gave good consistencies for the detection of sPLA2-IIA in serum samples.

Based on the calculated *t* values (Table 6), there are no significant differences between concentrations measured by both biosensor and ELISA immunoassay methods for the detection of sPLA2-IIA in human blood serum samples.

The bacterial sepsis biosensor offered important advantages such that: (i) It can analyze a large number of samples simultaneously; (ii) relatively short time is needed for result generation; (iii) high specificity and good sensitivity; (iv) high-throughput system and potential for semi automation; (v) high portability (can be used in disasters or remote areas when needed). Moreover, biosensor system for sepsis diagnosis will enable rapid diagnosis of bacterial sepsis accurately and enable physicians to immediately initiate appropriate antibiotic therapy. This invention can be used for detecting human sPLA2-IIA level rapidly at patient bedside to detect bacterial infection. The conventional way for detecting sPLA2-IIA using an ELISA assay is not user friendly and takes longer time for diagnosis. Results generated always tend to be inconclusive, hence this makes an ELISA protocol less ideal for sepsis diagnosis. In addition, the current ELISA kit available in the market is not licensed for diagnostic use. As far as our knowledge is concerned there is no specific diagnostic and prognostic kit available in the market to ‘clinically’ diagnose sepsis incidence. Our developed prototype will enable rapid bacterial sepsis diagnosis to be carried out, assisting physicians in risk stratification for effective patient management and improvement of clinical outcome. This will lead to shorter duration of hospitalizations, reduction of patient morbidity and mortality, contributing towards enhancing the quality of life for patients. Usage of this kit will not be limited to hospitals and medical fraternity, but also in the community (nursing homes, military, sports center, pharmacies, schools and private homes), revolutionizing infection diagnosis to become an accurate, rapid and commonly accessible process.

## 4. Conclusions

The electrochemical biosensor based on tri-enzyme system was successfully constructed for the detection of human sPLA2-IIA in serum samples in diagnosis of sepsis and bacterial infection. With the above results, sPLA2-IIa showed excellent performance and can be used as a versatile biomarker to diagnose both sepsis and bacterial infection, independently. It may assist in early antimicrobial administration and may be a useful tool, either alone or in combination with other markers in identifying sepsis and bacterial infection. The detection level of sPLA2-IIA may expedite the initiation of early antimicrobial therapy and sepsis bundle. The performances of enzyme biosensor demonstrated the linear response range was 0.01–100 ng/mL (*R*^2^ = 0.98304) with limit detection of 5 × 10^−3^ ng/mL. The proposed enzyme biosensor is comparable with the conventional method based on the ELISA immunoassay method for the evaluation of sPLA2-IIA in human serum samples. This point-of-care sepsis and bacterial infection diagnostic kit will assist the physician rapidly diagnose sepsis and bacterial infection worldwide.

## 5. Patents

This work has already been patented with Centre for Collaborative Innovation, Universiti Kebangsaan Malaysia (Patent number: PI2016001396 through ETP-2013-050).

## Figures and Tables

**Table 1:** The optimized amount of AuNPs and ACMS in the fabrication of the biosensor.

No.	Parameters	Value Range	Optimal Value
1	Amount of AuNPs (mg)	0.03–0.1	0.05
2	Amount of ACMS (mg)	0.02–0.2	0.04

**Table 2:** Percentage DPV peak current of sepsis biosensor compared with the metabolite compound i.e., urea, ascorbic acid, sucrose, glucose and citric acid.

Metabolite Compounds	DPV Peak Current (μA)	Sepsis Biosensor (%)	
		80 ng/mL	40 ng/mL
sPLA2-IIA	16.11 ± 1.02	100	100
Ascorbic acid	3.16 ± 0.41	19.6	18.4
Urea	1.83 ± 0.07	11.4	10
Sucrose	1.58 ± 0.02	9.8	9.5
Glucose	1.24 ± 0.03	7.7	6.5
Citric acid	0.93 ± 0.02	5.8	4.9

**Table 3:** The data of DPV peak current (take at 0.2 V) toward various concentrations of sPLA2-IIA verified by potassium ferricyanide, K_3_Fe(CN)_6_ as mediator.

Conc. (sPLA2-IIA)	Mean ± SD (Current, μA)	RSD (%, *n* = 3)
0.01	1.14 ± 0.053	4.673
0.10	1.88 ± 0.157	8.367
1	2.14 ± 0.049	2.287
10	3.09 ± 0.211	6.851
20	4.84 ± 0.509	10.525
40	6.95 ± 0.146	2.101
60	8.38 ± 0.684	8.162
80	9.94 ± 0.738	7.424
100	13.43 ± 0.882	6.567

**Table 4:** The data of DPV peak current (take at 0.2 V) toward various human serum samples for sPLA2-IIA detection verified by potassium ferricyanide (K_3_Fe(CN)_6_) as mediator.

Samples	Mean ± SD (Current, μA)	RSD (%, *n* = 3)	Plotted (ng/mL)	Elisa Method (ng/mL)
Healthy	1.934 ± 0.189	9.786	0.145	0.148
Sepsis	4.620 ± 0.465	10.061	24.349	24.30
Septic shock	5.551 ± 0.521	9.379	32.731	32.95

**Table 5:** The serum level of human sPLA2-IIA obtained from tri-enzyme bacterial sepsis biosensor for three samples of human serum.

Samples	sPLA2-IIA Level (ng/mL)	
	Mean ± SD	RSD (%, *n* = 3)
Healthy	0.145 ± 0.009	6.174
Sepsis	24.35 ± 2.457	10.090
Septic shock	32.73 ± 2.102	6.423

SD = standard deviation; RSD = relative standard deviation.

**Table 6:** Comparisons of the sepsis data measured with biosensor and ELISA immunoassay and verified with *t* test values.

Samples	Biosensor Method			ELISA Method	*t* Test
	Current (μA)	RSD (%, *n* = 3)	Human sPLA2-IIA (ng/mL)	Human sPLA2-IIA (ng/mL)	
Healthy	1.93 ± 0.19	9.78	0.145 ± 0.01	0.148 ± 0.01	0.117
Sepsis	10.06 ± 0.37	7.90	24.349 ± 2.46	24.303 ± 2.31	0.018
Septic shock	5.55 ± 0.42	7.57	32.732 ± 2.10	32.950 ± 3.37	0.091

Note: *t*-experiment, *t*_4_ = 2.78 (95%, *p* < 0.05), RSD = relative standard deviation.
